# Newgrowth in the Bladder*Read before the Buffalo Medical Association, July 1, 1879.

**Published:** 1879-08

**Authors:** Herman Mynter


### NEWGROWTH IN THE BLADDER OF A CHILD.*

BY HERMAN MYNTER, M. D.

Allow me, gentlemen, to present a singular case of newgrowth in the bladder, and to add to my report some remarks concerning this disease.

The patient, a little girl sixteen months of age, daughter of a clergyman in a neighboring village, is a strong, healthy child, well developed and well nourished. For about one year she has urinated very frequently, and, every time, she strained, screamed and kicked with all her might, for a short time after the urine had passed. Four weeks ago the mother discovered a little red, bleeding tumor passing out of the vagina during the straining. She brought the child to me the 11th of June of this year. On account of the violent resistance of the child, chloroform was necessary, having administered which, I examined the parts, Dr. Howe being present. Nothing was seen except a slight œdematous swelling of the hymen, but suddenly the child passed water, and, thereafter, strained with much force. Immediately a red,

* Read before the Buffalo Medical Association, July 1, 1879.

lobular, bleeding, pedunculated tumor, as large as a nut was pressed out through the hymen. I immediately applied a ligature around the base of this tumor, but this produced increased straining, and several smaller growths appeared on all sides of the larger one. Believing I had here a polypous newgrowth of the vagina, and as the larger tumor obstructed the view of the parts, I desisted, for the moment, from further examination. Three days afterward I again gave the child chloroform, Drs. Lothrop and Barker being present, and found that the ligated tumor had fallen off. A small speculum was then introduced into the vagina, and a little above the hymen, on the posterior vaginal wall, was found the rest of the severed pedicle in the form of a little granulating prominence. No trace was discovered of the other tumors, and I therefore believed that, without knowing it, I might have gotten the ligature around them too. The removal of the large tumor having had no effect whatever on the pain and frequency of urinating, I therefore introduced a sound into the bladder, and both Dr. Lothrop and myself noticed a click, as though the sound had touched a stone. Two days afterward I attempted to remove the stone, Drs. Tobie, Hopkins, Folwell, Cary and Barnes, being present. The child was again put under the influence of chloroform, but, having introduced the sound, I could not now get the click. At last I felt it distinctly; Dr. Tobie also felt it, and Dr. Cary, too, although the latter gentleman did not feel perfectly satisfied. He had felt something, but he thought there was some substance between the stone and the instrument. I gradually diluted the urethra with Gouley's instrument for dilating strictures, and at last introduced the forceps, but no stone could be found. I got something in the forceps every time, but it was adherent, and was not hard enough. I introduced my finger gradually, to explore the bladder, but could detect no stone. Believing the fragment to be so small as to escape detection, I syringed the bladder out with lukewarm water. The child immediately commenced to strain, and pressed out through the urethra a polypous mass as large as the egg of a pigeon, consisting of hundreds of small pedunculated tumors, the largest of which was the size of a bean, of an oval shape, hard as cartilage; and the smallest globular in form, as large as the head of a pin, and seemingly springing up from the mucous membrane everywhere. The tumors were of a pale color, and had a very deficient vascular supply. I took hold of a large part of this mass with the forceps, and gently tore it off, by turning the forceps; several others I cut off with the scissors. Very little bleeding occurred, but there seemed to be no end to the growths. The whole, considered as one tumor, had a broad base, consisting of the mucous membrane of the bladder, and it seemed to us that the whole interior surface of the bladder was involved in this singular newgrowth. The more we pulled the more came out; and, believing in this case discretion to be the better part of valor, I therefore, the other physicians concurring, reduced the tumor and cleaned the cavity of the bladder. The following days the child felt better, had no fever, and, as the water passed involuntarily, on account of the dilatation of the urethra, there was no straining and less pain than before the operation. Dr. Hopkins, who made a microscopical examination of the tumor, reports that it consisted of connective and fibrous tissue with very few cells, and was covered with normal epithelium. The newgrowth, therefore, must be considered as a fibrous tumor, or rather, as a collection of fibrous tumors.

This, gentlemen, is then a case of a polypus in the vagina in a little girl sixteen months old; and, what is worse, a newgrowth in the bladder, the growth, in all probability being a fibromatous polypus, benign in its character, but malignant by virtue of its position and the derangement to the functions of the urinary organs. Tumors in the bladder, according to Bilroth and Pitha, are found oftener in women than in men, are generally only found in old age, but may, exceptionally, be found in children. One case is mentioned in Bilroth and Pitha's surgery, being that of a female child under four years of age. They may be found anywhere in the bladder, but the places of preference are collum vesicæ and trigonum vesicæ. The tumors are generally cancers or sarcomas, but we sometimes find mucous polypi, cysts, fibrous tumors, and other forms. The newgrowths are partly primary, having started in the wall of the bladder (and these tumors are generally benign in their character, although cancers may take their starting point here), partly secondary, attacking the bladder from the neighboring organs.

In regard to symptoms, newgrowths, if they are small and not located in the neck of the bladder, often do not manifest themselves; if they are large, they may give origin to violent symptoms, as ischuria, dysuria, hydronephrosis, severe bleedings, cystitis, etc., etc. If incrustations occur, it may have every appearance of being a stone, as in the case above reported.

In regard to the diagnosis, I have very little to offer. It is evident that the diagnosis may be very difficult. In this case, where the tumor presented itself at last, it was very easy but until then we had no thought of a newgrowth here. That the tumor may simulate a stone is evident. It was our luck, in this case, that the patient was a little girl; for had it been a boy, I should probably, being so sure of having a stone, have made lithotomy. As it was, there was scarcely any operation, the child herself having dilated the urethra very much by continued straining and attempts to discharge the polypus.

Also in regard to prognosis, little can be said. It is evident that the most severe complications may arise, and that on the other side, a little polypus may be harmless if it has its position in the fundus of the bladder; but even if the tumors be of benign character, the prognosis is generally unfavorable, as it is very difficult to remove them.

The treatment is operative, but the operation is so difficult, and the chances of success so few that some surgeons advise not to touch them.

If we have a pedunculated tumor situated at the neck of the bladder in a female, we may try to tear it off, ligate it, cut it off, or crush it with instruments of various forms. The danger lies in the bleeding, and in the possible perforation of the bladder. It will be necessary in the majority of cases, first to dilate the urethra forcibly, which is easily done in case the patient is a female—in males, the operation must, necessarily, be more dangerous. Another way is to open the bladder, either through sectio alta above the symphysis, or by vestibular incision, or by vaginal incision (kolpo-cystotomy).

Liston removed a large cyst by sectio alta; Nusbaum more than once removed pieces of the wall of the bladder as large as a dollar, by operation for cancer of the rectum, and not even a fistula remained. But what is there to be done in a case like this, where the patient is sixteen months old, and the newgrowth is not pedunculated, but consists of a great many small tumors springing up from the mucous membrane? To remove the whole of the diseased membrane at once would scarcely be possible without injuring the bladder so much that death would be almost certain. To try, little by little, to cut off the tumors as they present themselves when the child strains seems almost a hopeless task, considering the great number of tumors of different sizes that we saw. To leave the child to its sufferings, which judging from its screams, must be considerable, seems cruel. What is then to be done? Considering that the child feels better when the urine passes involuntarily, would it not be advisable, if it shows itself to be impossible to remove the growths, gradually, to establish a vesico-vaginal fistula? I would suggest that as a means of relieving the child.

In the Philadelphia *Medical Times*, of July 5th, Dr. Lenox Hodge has an article on kolpo-cystotomia, which he recommends for irritable bladder or chronic cystitis. In chronic cystitis, the operation should be performed in order to give relief to the pain and straining, to prevent retention of urine, and to allow the bladder rest. The object is to leave an opening for a year or more through which the urine may flow instead of flowing along the urethra.

When the cause of the cystitis can be found and removed, as in retroflexion of the uterus, no one would think of performing this operation. But when the cause cannot be found, or if found, cannot be removed, or if, when removed, the pain and inflammatory symptoms continue, he thinks kolpo-cystotomy is indicated. Dr. Hodge mentions two cases, in the first of which the result, although not perfect, was favorable, in so far that the patient, who had been obliged to urinate with great pain from twenty to twenty-five times every night, was able after the operation, to remain in bed all night, and her pains were greatly diminshed. No diagnosis is given.

The diagnosis of the second case is still less clear. In neither case did the pain disappear entirely, which Dr. Hodge considers as a proof that the pain was reflectory and not solely due to the urine in the bladder, although the sensations of the patients seemed to indicate this. He therefore points out the importance of finding and removing the cause of reflex pain before resorting to kolpo-cystotomy. Secondly he mentions that all the urine did not escape involuntarily, but that, even after the operation, considerable straining was necessary. The cause of this was, in his opinion, that the presence of urine in the vagina produces spasmodic contraction and closure of the orifice of the vagina, and the only power of expulsion is in the abdominal muscles and diaphragm acting at a great distance and under a disadvantage. He therefore concludes that we must not expect to relieve all the pain, nor that the urine will pass out without any effort—facts which are of importance for the physician who contemplates such an operation.

